# Infestation of Field Dodder (*Cuscuta campestris* Yunck.) Promotes Changes in Host Dry Weight and Essential Oil Production in Two Aromatic Plants, Peppermint and Chamomile

**DOI:** 10.3390/plants9101286

**Published:** 2020-09-29

**Authors:** Marija Sarić-Krsmanović, Ana Dragumilo, Jelena Gajić Umiljendić, Ljiljana Radivojević, Ljiljana Šantrić, Rada Đurović-Pejčev

**Affiliations:** 1 Institute of Pesticides and Environmental Protection, Banatska 31b, 11080 Belgrade, Serbia; pecikos@gmail.com (J.G.U.); ljiljana.radivojevic@gmail.com (L.R.); ljiljkasantric@gmail.com (L.Š.); rajcica76@gmail.com (R.Đ.-P.); 2 Institute for Medicinal Plant Research “Dr. Josif Pančić”, Tadeuša Košćuška 1, 11000 Belgrade, Serbia; anamatkovic89@gmail.com

**Keywords:** parasitic plant, essential oil, *Mentha piperita*, *Chamomilla recutita*

## Abstract

Peppermint (*Mentha piperita* L.) and chamomile (*Chamomilla recutita* (L.) Rausch.) are aromatic plants with considerable economic value. These plants and their essential oils are used in medicine, cosmetics, and the food industry. One of the main limiting factors in peppermint and chamomile commercial cultivation is weed competition since weeds are able to decrease both oil amount and biomass yield. The purpose of the present study was to determine the effect of parasitism by field dodder (*Cuscuta campestris* Yunck.) on peppermint and chamomile dry weight and their essential oil yield and composition. Essential oils from both noninfested and infested peppermint and chamomile plants were obtained by hydrodistillation and characterized chemically by gas chromatography (GC) coupled with mass spectrometry (MS). The amount of dry matter accumulated by peppermint and chamomile plants infested by field dodder was lower (25% and 63%, respectively) compared to noninfested plants. Essential oil yield increased for peppermint (3.87% (*v*/*w*) and 3.63% (*v*/*w*)), but decreased for chamomile (0.2% (*v*/*w*) and 0.5% (*v*/*w*)) both from infested and noninfested plants, respectively. The oil composition profile significantly differed in terms of content. In peppermint plants, field dodder infestation increased menthone content by 23%, and decreased the content of both menthol by 11% and pulegone by 67%. Furthermore, δ-cadinene was detected only in oil extracted from infested peppermint plants. Compared to peppermint, chamomile plants were significantly more affected by field dodder in terms of essential oil yield, as well as oil composition and plant dry weight. In chamomile plants, (*E*)-dendrolasin was detected in the oil of noninfested plants, and 1,4-dimethyl-7-(1-methylethyl)-azulen-2-ol was detected only in the oil of infested plants.

## 1. Introduction

Plant secondary metabolites play a recognized role in interactions between plants and their environment and they also act as defense chemicals [1]. Production of these compounds is very low (less than 1% dry weight) and mainly depends on the physiological and developmental stage of plants [2,3]. Production and accumulation of metabolites by plants of the same species growing in different regions may be affected by environmental factors, so that such substances appear to act as a chemical interface between the plant and the surrounding environment [1,4,5]. Although secondary metabolites of medicinal and aromatic plants are commonly generated through genetic processes, their biosynthesis depends to a great extent on environmental factors, both abiotic (soil type, water availability, nutrient solubility, light, UV radiation, location, edaphic and seasonal variation, etc.) and biotic, which affect growth parameters, essential oil yield and oil constituents [6,7,8,9,10]. Pirzard et al. [11] indicated that irrigation regimes and field capacity affected the yield of dry chamomile flowers and essential oil percent and its composition. Šalamon et al. [12] showed a strong impact of edaphic and seasonal variations on the composition of essential oil from chamomile originating from various parts of Iran. Santoro et al. [13] revealed that some vegetative parameters (shoot and root lengths, shoot fresh weight, root dry weight, etc.) of peppermint increased upon exposure to volatile organic compounds from two different plant growth-promoting rhizobacteria (*Pseudomonas fluorescens* and *Bacillus subtilis*). Besides, the authors showed that rhizobacterial volatile organic compounds impacted the formation of plant secondary compounds, finding a two-fold increase in the accumulation of essential oil from peppermint plants treated with *P. fluorescens,* compared to *B. subtilis.* Guo et al. [14] revealed some beneficial effects of colonization of peppermint roots by the fungus *Trichoderma viride* as it elevated essential oil yield and composition.

Peppermint and chamomile are medicinal and aromatic plants that have long been grown for their essential oils as valued aromatic agents. They are highly regarded and widely used medicinal plants in traditional medicine and grown for their dry flowers and leaves that are used for herbal teas. Peppermint (*Mentha piperita* L.) is a hybrid mint, a cross between *Mentha aquatica* L. and *Mentha viridis* L. It is a perennial species of the family Lamiaceae cultivated mainly as an annual crop. The content and composition of peppermint essential oil have economic value for producers, processing industries, manufacturers of peppermint-containing products, as well as for consumers [15]. Literature sources reveal that the dry aerial biomass of peppermint growing in Serbian localities contains approximately 1–2% essential oil, while leaves contain 2–4%, including approximately 40 chemical components in it, the major of which are: menthol, menthone, iso-menthone, limonene, neo-menthol, menthofuran, trans-sabinene hydrate, pulegone, menthyl acetate and β-caryophyllene [16]. Chamomile (*Chamomilla recutita* (L.) Rausch., syn. *Matricaria chamomilla* L.) is a long-known medicinal plant species of the family Asteraceae, which has often been referred to as the “star of medicinal species”. Chamomile flowers grown for drugs have approximately 120 chemical constituents, such as terpenoids and flavonoids, which have anti-inflammatory, antiseptic, stimulating, carminative, spasmolytic, and sedative activity. Chamomile flowers produce blue essential oil whose yield varies from 0.2 to 1.9%, while their leading constituents are α-bisabolol, chamazulene and farnesene [12,17,18].

Besides the mentioned abiotic factors that can remarkably affect the chemical constituents of an aromatic plant, one of the main limiting factors in peppermint and chamomile commercial cultivation is weed competition [19] as weeds are able to reduce both oil amount and biomass yield. Dhima et al. [20] reported that the largest losses in annual cultivated aromatic plants were caused by weeds. Matković et al. [21] recorded a 88% loss of aerial biomass of peppermint to weeds, compared to non-weeded control in South Banat in Serbia. Darre et al. [22], in two-year trials, revealed a yield loss of the same species of 35.7% and 50.9% in weeded, compared to non-weeded plots at a Cordoba site in Argentina. Similarly, Karkanis et al. [23] reported a significant yield loss in peppermint fields heavily infested with perennial weeds. Autotrophic flowering plants are predominant among weed species, but weeds also include parasitic plants that have developed various ways of attacking their hosts, by using a special adhesion/absorption organ known as the haustorium. The organ serves as a structural and physiological bridge for parasites to draw water, minerals, organic molecules and solutes from the host plant conductive system, leading to severe host growth and yield reductions [24,25,26]. Dodder are parasitic weeds species belonging to the *Cuscuta* genus. Among them, field dodder (*Cuscuta campestris* Yunck.) is one of the most widespread species, which infests a wide range of crop species [27], inducing negative impacts on the growth and yield of infested hosts, and it has significant effects on the structure and functioning of plant communities that are infested by these holoparasites [28,29,30]. Field dodder prefers a variety of forage crops, vegetables, and ornamental and aromatic plants (including peppermint) that are rich in volatile compounds [31].

To the best of our knowledge, there are currently no other reports on the effects of field dodder on the dry weight of aerial biomass and yield and on the main constituents of the essential oils of peppermint and chamomile plants. So, the objective of this study was to evaluate the effects of field dodder on the yield, composition and contents of main components of the essential oils and dry weight of peppermint and chamomile plant biomass.

## 2. Results and Discussion

### 2.1. Effects of Field Dodder on Peppermint Aerial Biomass and Oil Production

The present study revealed a strong negative impact of field dodder infestation on peppermint aerial dry weight (Table 1). The dry weight of aerial biomass of noninfested peppermint plants (1.24 g ± 0.09) was 1.3-fold higher than the dry weight of infested plants (0.94 g ± 0.08), while the dry weight biomass of field dodder was 46.68 g ± 0.51.

Despite the reduction in aerial biomass of infested peppermint plants, field dodder increased the concentration of oil in them from 3.63% (*v*/*w*) in noninfested to 3.87% (*v*/*w*) in infested plants (Table 2). Our previous research [32] offers confirmation of this observation as field dodder infestation increased the yield of essential oils in fennel plants from 5.6% (*v*/*w*) in noninfested plants to 5.8% (*v*/*w*) in infested plants.

Chemical compositions of the essential oils of noninfested and infested peppermint plants are presented in Table 3. Chromatograms are reported in Supplementary Materials (Figures S1 and S2). In both oils, from infested and noninfested plants, 38 identified components accounted for 99.35 and 99.45% (*v*/*w*) of total mass, respectively. Considering the yields (%, *v*/*w*) (Table 2), and density (0.90 and 0.91 g/mL) of essential oils from infested and noninfested plants, the identified compounds made up 3.46% (*v*/*w*) and 3.29% (*v*/*w*) of infested and noninfested plant biomass used for essential oil isolation, respectively. Menthone and menthol were the main components of peppermint essential oil, regardless of the field dodder infection status. Menthone content increased by 23%, while menthol content decreased by 11% in the oil extracted from infested peppermint plants, as compared to noninfested plants. Other oil components that were reduced in peppermint by field dodder infestation were (*E*)-β-ocimene by 42% and γ-terpinene by 38%. In contrast, field dodder infestation increased the content of some oil components, not only menthone but also trans-sabinol, piperitone, δ-selinene and viridiflorol (Table 3). There was no significant difference in the contents of sabinene, myrcene, 4-terpineol, β-caryophyllene and caryophyllene oxide. On the other hand, a minor δ-cadinene level (0.09%) was only detected in the essential oil of infested peppermint. Essential oils of peppermint originating from Serbia usually contain δ-cadinene as a component [33,34]. Considering the very low δ-cadinene level (0.09%), it seems that peppermint plants do not metabolize any additional components in response to the presence of field dodder. In addition, the essential oil of infested peppermint plants (Table 3) did not contain compounds previously reported for different *Cuscuta* species (vanillin, eugenol, α-cadinene) [35,36], or nonanal as a compound involved in chemical defense and one responsible for host colonization by *Cuscuta* species [37].

Regardless of the status of peppermint plants, the contents of the predominant oxygenated monoterpenes (Table 3) were high and very similar (84.18 and 83.54% in infested and noninfested plants, respectively). The contents of other chemical classes were also similar. Thus, monoterpene hydrocarbons accounted for 3.81 and 4.25% of infested and noninfested peppermint plants, respectively, while sesquiterpene hydrocarbons represented 4.44 and 4.14%, and oxygenated sesquiterpenes accounted for 1.04 and 0.97%, respectively. All other compounds made 0.60 and 0.47% of the noninfested and infested oils, respectively (Table 3).

A comparison of composition data for peppermint essential oils obtained from previous reports in Serbia and our present findings revealed a fairly good consistency. Menthone and menthol were generally found to be the main components of peppermint essential oils [39,40], while the peppermint oils were also rich in menthyl acetate in previous experiments in Serbia [33,34,41], but were not abundant in the plants of our present research regardless their field dodder infestation status. Field dodder infestation affected the content of other minor oil constituents, reducing the content of pulegone by 67%. Contrary to our findings, Santoro et al. [13] revealed that pulegone was 30% higher in the essential oil of peppermint plants exposed to volatile organic compounds from *P. fluorescens*. Furthermore, these authors found that menthofuran was reduced to 0.8%, as compared to 1.16% in controls, while in the present study, the content of this compound increased by 5% in the oil extracted from field dodder-infested peppermint plants. Guo et al. [14] showed that colonization of *Trichoderma viride* fungi in peppermint roots had an impact on the composition of essential oil, increasing the percentage of menthol, menthone, and pulegone, and decreasing menthofuran compared to control plants. Sharma et al. [42] revealed alterations in the lipid composition in host plants (*Medicago sativa* L., *Helianthus annuus* L., *Pisum sativum* L. and *Lantana camara* L.) upon infestation by *Cuscuta reflexa*. Dodder infestation significantly increased the total lipid level of the hosts and the increase was mainly due to enhancement in the neutral lipid fraction, while the total phospholipid levels were not affected as a result of the infestation by this parasitic weed. In addition, Mishra and Sanwal [43] revealed changes in the lipid composition of susceptible varieties of *Brassica juncea* seed oil upon infection by *C. reflexa*. The infestation by dodder resulted in a decrease in oil content and the composition of the oil of susceptible mustard varieties became poorer. A significant increase in free fatty acids and free sterols and decrease in total neutral glycerides of oil were observed. Sarić-Krsmanović et al. [32] showed that field dodder infestation affected the main components of the essential oil isolated from fennel plants by reducing the content of trans-anethole by 67.0% and increasing the content of fenchone by 26.5%, and methyl chavicol by 19.6%.

### 2.2. Effects of Field Dodder on Chamomile Aerial Biomass and Oil Production

In chamomile plants, dry biomass weight and essential oil yield were more affected by field dodder infestation than in peppermint plants (Table 1 and Table 2). The dry weight biomass of field dodder plants was observed as 39.49 g ± 0.48, which induced a 2.7-fold reduction in aerial biomass in chamomile plants from 0.83 g ± 0.06 in noninfested plants to 0.31 g ± 0.05 in infested plants. The obtained data showed that the essential oil yield from noninfested plants (0.5% *w*/*v*) was 2.5-fold higher than it was from infested plants (0.2% *w*/*v*), which clearly indicates a strong influence of field dodder on the composition of chamomile secondary metabolites studied (Table 2). Different host plant responses to field dodder as a parasite may be partially associated with tendencies associated with plant stress response of different plant taxa, and potentially suggest some unknown stress response mechanisms in host plants [44]. Parasitic plants inhibit host growth and reproduction by capturing their nutrients, and so disturbing their balance of resources [28]. Differences in infestation levels of different hosts by the same *Cuscuta* sp. may result from their different nutrient status or their size (metabolic activities) [45]. The parasitic genus *Cuscuta* is known to constitute a strong competitive sink as its species divert a large portion of photoassimilates of their hosts into their tissues [29,46,47,48]. However, Sarić-Krsmanović et al. [26] revealed higher contents of some nutrients in infested than in noninfested alfalfa and sugar beet plants. A final assessment (40 days after infestation) revealed that field dodder raised the contents of N, P2O5, K2O and organic nutrients in infested alfalfa plants, while infested sugar beet plants had higher contents of N and organic nutrients than noninfested plants. Similarly, *Cuscuta reflexa* infestation results in stimulated host nitrate uptake, and higher rates of photosynthesis in both young and mature leaves compared with control plants [49,50]. Field dodder also had a strong impact on pigment contents (chlorophyll *a* and *b*, carotenoids) in alfalfa and sugar beet plants [26]. Similarly, Sarić-Krsmanović et al. [51] confirmed that this parasitic plant also affected several parameters of chlorophyll fluorescence (minimal fluorescence, variable/maximal fluorescence ratio, effective fluorescence yield, variable fluorescence, and intensity of fluorescence).

The main components of the essential oils isolated from chamomile grown alone and infested with field dodder are presented in Table 4. Chromatograms are shown in Supplementary Materials (Figures S3 and S4). Twenty-three components were identified in both essential oils, and they accounted for 99.91 and 99.90% (*v*/*w*) of total oil obtained from noninfested and infested plants, respectively. Considering oil density (0.91 g/mL for both oil) and yields (%, *v*/*w*) (Table 2), the identified compounds made up 0.45 and 0.18% (*w*/*w*) of noninfested and infested chamomile biomass used for essential oil isolation, respectively. With an exception of (*E*)-dendrolasin, which was detected only in noninfested chamomile essential oil, and 1,4-dimethyl-7-(1-methylethyl)-azulen-2-ol found only in oil extracted from infested chamomile, all other components were identified in both oils tested. These results indicate that chamomile plants metabolize some different components, depending on field dodder infestation. In this regard, literature data on the chemical composition of chamomile essential oils originating from Serbia vary considerably, as well as in comparison with our present findings. For example, chamomile oils have been reported to be mainly rich in (*E*)-β-farnesene [52], as well as α-bisabolol oxide A and iso-aromadendrene epoxide [53], and α-bisabolene oxide A and (*Z*)-en-yn-dicycloether [54]. Differences are probably due to different geo-climatic conditions in those studies. Šalamon et al. [12] analyzed essential oils from chamomile originating from various parts of Iran and found that the plants were grown under conditions of mild winters, very humid and hot summers and produced oil rich in α-bisabolol, while those grown under conditions of arid weather had a high content of α-bisabolol oxide A.

The main components of oils from both infested and noninfested plants in our study (Table 4) were α-bisabolol oxide B and α-bisabolol oxide A, followed by α-bisabolene oxide A and chamazulene. The contents of both α-bisabolol oxides and α-bisabolene oxide were higher in infested plants, while noninfested plants were richer in chamazulene. Field dodder infestation also affected the content of other minor oil constituents, reducing the content of (*Z*)-en-yn-dicycloether by 84%. Other oil components that were reduced by field dodder infestation were (*E*)-en-yn-dicycloether (by 56%), β-selinene (by 54%), and (*E*)-β-farnesene (by 49%). In contrast, field dodder infestation increased the content of some oil components besides α-bisabolol oxide A and B and α-bisabolene oxide A, namely guaia-6,10(14)-dien-4β-ol and salvia-4(14)-en-1-on (Table 4). There was no significant difference only in the content of nerolidol oxide. Furthermore, sesquiterpene compounds accounted for more than 87% of total oil mass of both oils, while oxygenated sesquiterpenes represented 74.11 and 87.24% of noninfested and infested oil mass, respectively, followed by 13.71 and 8.77% of sesquiterpene hydrocarbon, respectively.

Field dodder affects the growth and reproduction of its hosts more or less severely, depending on the infested plant species. Stressed plants may be surprisingly good hosts, even when they are small, because they have metabolite reserves that are more concentrated and not allocated to growth, and also due to their accumulation of additional carbon- and nitrogen-containing compounds [55]. The changeable yield and main components of essential oils from infested peppermint and chamomile plants, compared to noninfested, may therefore be considered as the host response to parasitism, which mostly leads to the accumulation of nutrients as heightened metabolism creates a defense mechanism in the host.

## 3. Materials and Methods

### 3.1. Study Site

Peppermint (*M. piperita*) and chamomile (*C. recutita*) plants were grown in fields of the Institute for Medicinal Plant Research “Dr. Josif Pančić”, Pančevo, South Banat, Serbia (44°52′28.4′′ N 20°41′56.2′′ E and 44°52′21.9′′ N 20°41′50.1′′ E, respectively). Peppermint stolons were hand transplanted in the autumn of 2016 in rows, and at 10 cm depth, and 70 cm spacing. The stolons originated from a large-scale peppermint production unit at the Institute. Chamomile was sown at the beginning of October 2016 and at a sowing rate of 2–3 kg/ha. Winter wheat (*Triticum vulgare* Vill.) was the pre-crop of both plants and fertilization was applied immediately after deep autumn plowing (November 2016) using compost (25 t/ha). The soil type was chernozem with 2.3% humus, while total nitrogen content was 0.19%. The pH was 6.4 in H_2_O, and 5.4 in KCl. Ammonium lactate-extractable (available) P_2_O_5_ and K_2_O contents were 36 mg kg^−1^ and 362 mg kg^−1^, respectively. Irrigation was also applied three times during the growing season and in the amount of 20 L/ha.

The main weed species in the peppermint field were the annuals *Artemisia vulgaris* L., *Chenopodium album* L., *Consolida regalis* S.F.Gray, *Hibiscus trionum* L., *Senecio vernalis* W. et K., *Veronica hederifolia* L. and *Veronica persica* Poir. and perennials *Convolvulus arvensis* L. and *Sorghum halepense* (L.) Pers. The most abundant species was *C. album* with more than 8 plants/m^2^. The other weeds were counted in fewer than 5 plants/m^2^. In the chamomile field, the main weed species were the annuals *Anthemis arvensis* L., *Chenopodium album* L., *Consolida regalis* S.F.Gray, *Fumaria officinalis* L., *Galium aparine* L., *Senecio vernalis* W. et K. and *Veronica persica* Poir., and the perennials *Agropyrum repens* (L.) Beauv., *Cirsium arvense* (L.) Scop. and *Sorghum halepense* (L.) Pers. The most abundant species was *A. arvensis* with more than 12 plants/m^2^. The other weeds were counted in fewer than 4 plants/m^2^. In the spring of 2017, the spontaneous appearance of field dodder seedlings was also noted in both fields. Additional tests (seedling emergence) revealed that field dodder seeds were introduced into the fields with compost. Over the next few weeks, field dodder infestation was monitored. The autotrophic weeds, in stage from cotyledons to 2–4 true leaves, were manually removed from all plots in the peppermint and chamomile fields. Weed management is important because weeds and crops grow under competition for light, space, soil moisture and nutrients. In addition, the presence of autotrophic weeds in crops infested with field dodder can increase the degree of infestation with this parasitic plant, playing the role of intermediated host. Therefore, manual weeding was extended until the end of the experiment.

### 3.2. Collection and Preparation of Plant Material

The experiment was set up over an area of 500 m^2^ for each plant species and it included the following variants: Np—noninfested peppermint plants (control); Ip—infested peppermint plants; Nc—noninfested chamomile plants (control); Ic—infested chamomile plants. There were five plots in each variant and the plot size was 6.25 × 4 m (25 m^2^), while the distance between plots was 2 m. A wooden quadrat frame (50 cm × 50 cm) was used for sampling. Ten quadrats (two per plot) were sampled for each variant of the experiment. Samples collected from all 10 quadrats were added together and considered to constitute a representative sample of the community.

The samples of aromatic plants were collected at the flowering stages as follows: chamomile at the fully flowering stage (65 BBCH, 4th week of May 2017) while field dodder was at the vegetative growing stage; peppermint in the early flowering stage (60–61 BBCH, 3rd week of July 2017) while field dodder was at the flowering stage. The density of peppermint was 4–5 plants per quadrat frame. The density of chamomile was 25–28 plants per quadrat frame. Field dodder was widespread in small oases and aggregated on stems and leaves of peppermint plants, and on stems and leaf petioles of chamomile plants, forming a cover over the aromatic plants. Measurements relating to parasitization percentage of field dodder were carried out in the field plots. Based on visual assessment (percentage of area in trial covered by field dodder), the infestation was moderate, in chamomile plots by 40%, while peppermint plots were infested by 55%.

Plants from each quadrat were cut out just above the ground and samples were brought to the laboratory. In the next step, field dodder plants were manually and completely removed from the stems and leaves of infested plants. Fresh plant material was placed in thin layers in the shade of a draughty place, for the material to dry at the temperature of 20–22 °C for 30 days until reaching humidity levels of 10–12%. After being dried, each individual biomass of infested and noninfested plants was measured, and expressed as means in g/plant. Dry plant material was packed in paper bags and kept in a dry and cool place. After field dodder was separated from the stems and leaves of infested plants, the samples were placed in a drying oven at 60 °C for 72 h until a constant weight was reached. The dry weight of plant biomass was measured and expressed as means in g/m^2^.

### 3.3. Isolation of Essential Oil

Air-dried plant material without stems was subjected to hydrodistillation for 2.5 h, in a Clevenger type device, according to the standard procedure described in the European Pharmacopoeia [56].

### 3.4. Gas Chromatography (GC) Analyses and Identification of Components

Chemical composition of the tested essential oils was analyzed by a Varian CP-3800 gas chromatograph (GC) equipped with a split/splitless injector, DB-5MS column (30 m, 0.25 mm i.d., 0.25 µm film thickness), and Saturn 2200 mass spectrometer (MS) as a detection device. Injector temperatures were set to 250 °C, and the ion trap and transfer line temperatures were set to 250 and 280 °C, respectively. Helium was used as the carrier gas and its flow rate was 1 mL/min. The column temperature was linearly programmed to rise from 50 to 250 °C at a 4 °C/min rate before holding for 15 min. The mass detector was operated in the electron impact (EI) mode (70 eV; 40–600 *m*/*z* range). The essential oil solutions in n-hexane (1%) were injected in the split mode (1:20).

To determine the retention indices (RI), a mixture of n-alkanes (C6-C28) was analyzed by the GC—MS under identical conditions as the essential oils. Identification of the essential oil components was performed using both the Wiley 7.0 mass spectral library and the obtained RI data, while quantitative data were expressed as area percent obtained by the GC—MS analysis. The obtained RI data were compared to those in available literature [38]. The data were used as an additional tool to confirm MS findings.

### 3.5. Statistical Analysis

The biological experiment was performed in 10 replicates (*n* = 10). The number of injections on the GC-MS was performed in triplicate (*n* = 3). Data are expressed as means ± standard error. The data were processed in Statistika 8.0. software, using a one-way factorial analysis of variance (ANOVA), and the differences between noninfested and infested peppermint and chamomile plants were tested by Fisher’s Least Significant Difference (LSD) test (*p* < 0.05).

## 4. Conclusions

Chemical analyses revealed significant differences in the content of individual components in the tested peppermint (*M. piperita*) and chamomile (*C. recutita*) essential oils from noninfested plants and those infested with field dodder (*C. campestris*). These findings are also supported by differences in the obtained essential oil yields and biomass dry weight of peppermint and chamomile plants. Compared to peppermint, chamomile plants were significantly more affected by field dodder in terms of essential oil yield, as well as the composition and dry weight of its biomass.

The obtained data provide a basis for further studies aiming to examine chemically-mediated interactions between dodder and its aromatic plant hosts (peppermint and chamomile), and to establish whether dodder parasitism induces changes in other chemical constituents (phytohormones, volatiles or phenolics).

## Figures and Tables

**Table 1:** Aerial dry weight biomass of aromatic plants and field dodder.

Host	Treatment	Aromatic Host Aerial Biomass Dry Weight (g/plant ± s.e.)	Field Dodder Aerial Biomass Dry Weight (g/m^2^ ± s.e.)
Peppermint	Np (control)	1.24 ± 0.09 ^a^	-
	Ip	0.94 ± 0.08 ^b^	46.68 ± 0.51
Chamomile	Nc (control)	0.83 ± 0.06 ^a^	-
	Ic	0.31 ± 0.05 ^b^	39.48 ± 0.48

Np—noninfested peppermint plants (control); Ip—infested peppermint plants; Nc—noninfested chamomile plants (control); Ic—infested chamomile plants. Data are reported as the mean ± standard error (s.e.). Differences between dry weights of infested and noninfested aromatic plants biomass were evaluated by one-way analysis of variance (ANOVA) completed with Fisher’s Least Significant Difference (LSD) test; Means marked by different letters (a, b) differ significantly (*p* < 0.05).

**Table 2:** Yields of essential oils obtained from peppermint and chamomile.

Yield of Essential Oils (%, *v*/*w*)			
Peppermint		Chamomile	
Np	Ip	Nc	Ic
3.63	3.87	0.5	0.2

Np—noninfested peppermint plants (control); Ip—infested peppermint plants; Nc—noninfested chamomile plants (control); Ic—infested chamomile plants.

**Table 3:** Chemical compositions of peppermint essential oils (noninfested and infested plants).

N^0^	Components	RI_EXP_^1^	RI_LIT_^2^	Class ^3^	Content (%)		Infested Peppermint/Noninfested Peppermint (Ratio)
					Noninfested Peppermint (Control)	Infested Peppermint	
1	α-thujene	925	924	MH	0.07 ± 0.01 ^a^	0.06 ± 0.01 ^b^	0.86 *
2	α-pinene	932	932	MH	0.66 ± 0.03 ^a^	0.62 ± 0.02 ^b^	0.94 *
3	sabinene	972	969	MH	0.61 ± 0.02 ^a^	0.59 ± 0.04 ^a^	0.97 ns
4	β-pinene	975	974	MH	1.21 ± 0.11 ^a^	1.14 ± 0.08 ^b^	0.94 *
5	myrcene	988	988	MH	0.31 ± 0.02 ^a^	0.30 ± 0.02 ^a^	0.97 ns
6	3-octanol	992	988	O	0.22 ± 0.01 ^a^	0.18 ± 0.01 ^b^	0.82 *
7	p-cymene	1023	1020	MH	0.69 ± 0.04 ^a^	0.64 ± 0.03 ^b^	0.93 *
8	limonene + 1,8-cineole	1027/1029	1024/1026	MH/OM	5.85 ± 0.12 ^a^	5.51 ± 0.09 ^b^	0.94 *
10	(*Z*)-β-ocimene	1034	1032	MH	0.42 ± 0.04 ^a^	0.29 ± 0.01 ^b^	0.69 *
11	(*E*)-β-ocimene	1044	1044	MH	0.12 ± 0.01 ^a^	0.07 ± 0.01 ^b^	0.58 *
12	γ-terpinene	1056	1054	MH	0.16 ± 0.02 ^a^	0.10 ± 0.01 ^b^	0.63 *
13	*cis*-sabinene hydrate	1065	1065	OM	2.27 ± 0.18 ^a^	2.42 ± 0.10 ^b^	1.07 *
14	linalool	1096	1095	OM	0.38 ± 0.02 ^a^	0.40 ± 0.03 ^b^	1.05 *
15	(2*E*,4*E*)-octadienal	1104	1102	O	0.15 ± 0.01 ^a^	0.10 ± 0.01 ^b^	0.67 *
16	*trans*-sabinol	1138	1137	OM	0.10 ± 0.01 ^a^	0.12 ± 0.02 ^b^	1.20 *
17	*trans*-dihydro-α-terpineol	1143	1143	OM	0.12 ± 0.02 ^a^	0.13 ± 0.01 ^b^	1.08 *
18	menthone	1153	1148	OM	31.15 ± 0.35 ^a^	38.18 ± 0.56 ^b^	1.23 *
19	menthofuran	1162	1159	OM	8.82 ± 0.14 ^a^	9.28 ± 0.08 ^b^	1.05 *
20	menthol	1172	1167	OM	29.15 ± 0.18 ^a^	25.94 ± 0.13 ^b^	0.89 *
21	4-terpineol	1176	1174	OM	1.77 ± 0.04 ^a^	1.80 ± 0.03 ^a^	1.02 ns
22	*cis*-pinocarveol	1181	1182	OM	0.51 ± 0.03 ^a^	0.39 ± 0.02 ^b^	0.77 *
23	α-terpineol	1188	1186	OM	0.59 ± 0.03 ^a^	0.65 ± 0.03 ^b^	1.10 *
24	pulegone	1237	1233	OM	3.84 ± 0.07 ^a^	1.28 ± 0.04 ^b^	0.33 *
25	piperitone	1252	1249	OM	0.22 ± 0.01 ^a^	0.33 ± 0.01 ^b^	1.50 *
26	neo-menthyl acetate	1271	1271	OM	0.33 ± 0.02 ^a^	0.25 ± 0.01 ^b^	0.76 *
27	menthyl acetate	1290	1294	OM	4.12 ± 0.09 ^a^	2.90 ± 0.04 ^b^	0.70 *
28	iso-menthyl acetate	1305	1304	OM	0.17 ± 0.01 ^a^	0.11 ± 0.01 ^b^	0.65 *
29	(3*Z*)-hexenyl-(3*Z*)-hexenoate	1383	1383	O	0.23 ± 0.01 ^a^	0.20 ± 0.02 ^b^	0.87 *
30	β-bourbonene	1389	1387	SH	0.55± 0.02 ^a^	0.64 ± 0.04 ^b^	1.16 *
31	β-caryophyllene	1418	1417	SH	2.10 ± 0.03 ^a^	2.11 ± 0.06 ^a^	1.01 ns
32	α-humulene	1451	1452	SH	0.32 ± 0.02 ^a^	0.29± 0.02 ^b^	0.91 *
33	germacrene D	1479	1484	SH	0.96 ± 0.04 ^a^	1.01± 0.05 ^b^	1.05 *
34	δ-selinene	1495	1492	SH	0.21 ± 0.01 ^a^	0.30 ± 0.02 ^b^	1.43 **
35	δ-cadinene	1522	1522	SH	nd	0.09± 0.01 ^a^	- *
36	caryophyllene oxide	1582	1582	OS	0.37 ± 0.02 ^a^	0.36 ± 0.02 ^a^	0.97 ns
37	globulol	1589	1590	OS	0.35 ± 0.02 ^a^	0.37 ± 0.01 ^b^	1.06 *
38	viridiflorol	1598	1592	OS	0.25 ± 0.01 ^a^	0.31 ± 0.02 ^b^	1.24 *
	Total				99.35	99.45	1.00 ns
	Monoterpene hydrocarbons				4.25	3.81	0.90 *
	Oxygenated monoterpenes				83.54	84.18	1.01 ns
	Sesquiterpene hydrocarbons				4.14	4.44	1.07 *
	Oxygenated sesquiterpenes				0.97	1.04	1.07 *
	Others				0.60	0.48	0.80 *

^1^ RI_E_—Experimentally determined Retention Indexes; ^2^ RI_LIT_—Retention Indexes—literature data [38]; ^3^ Chemical class: MH—Monoterpene hydrocarbons, OM—Oxygenated monoterpenes, SH—Sesquiterpene hydrocarbons, OS—Oxygenated sesquiterpenes, O—Others; nd: not detected. Data are reported as the mean ± standard error (*n* = 3). Differences were evaluated by one-way analysis of variance (ANOVA) completed with Fisher’s Least Significant Difference (LSD) test; * *p* < 0.05. Means in the same row marked by different letters (a, b) differ significantly (*p* < 0.05); ns: not significant.

**Table 4:** Chemical compositions of chamomile essential oils (noninfested and infested plants).

N^0^	Components	RI_EXP_^1^	RI_LIT_^2^	Class ^3^	Content (%)		Infested Chamomile/Noninfested Chamomile (Ratio)
					Noninfested Chamomile (Control)	Infested Chamomile	
1	(*E*)-anethole	1281	1282	PP	0.19 ± 0.01 ^a^	0.21 ± 0.01 ^b^	1.11 *
2	(*E*)-β-farnesene	1452	1454	SH	2.75 ± 0.06 ^a^	1.41 ± 0.05 ^b^	0.51 *
3	dehydro-sesquicineole	1465	1469	OS	0.97 ± 0.04 ^a^	0.71 ± 0.03 ^b^	0.73 *
4	γ-muurolene	1479	1478	SH	0.16 ± 0.01 ^a^	0.12 ± 0.01 ^b^	0.75 *
5	germacrene D	1485	1484	SH	0.11 ± 0.01 ^a^	0.15 ± 0.01 ^b^	1.36 *
6	β-selinene	1491	1489	SH	0.24 ± 0.02 ^a^	0.11 ± 0.01 ^b^	0.46 *
7	(*E*)-nerolidol	1563	1561	OS	0.78 ± 0.04 ^a^	0.85 ± 0.04 ^b^	1.09 *
8	(*E*)-dendrolasin	1570	1570	OS	0.25 ± 0.01 ^a^	nd	- *
9	caryophyllene oxide	1583	1582	OS	1.28 ± 0.07 ^a^	0.74 ± 0.03 ^b^	0.58 *
10	salvia-4(14)-en-1-on	1593	1594	OS	0.15 ± 0.01 ^a^	0.24 ± 0.02 ^b^	1.60 *
11	guaia-6,10(14)-dien-4β-ol	1609	1610	OS	0.12 ± 0.01 ^a^	0.20 ± 0.01 ^b^	1.67 *
12	iso-aromadendrene epoxyde	1614	1612	OS	1.47 ± 0.07 ^a^	1.36 ± 0.09 ^b^	0.93 *
13	helifolen-12-al	1618	1619	OS	0.35 ± 0.02 ^a^	0.22 ± 0.01 ^b^	0.63 *
14	iso-spathulenol	1632	1630	OS	0.23 ± 0.01 ^a^	0.15 ± 0.01 ^b^	0.65 *
15	nerolidol oxide	1638	1640	OS	0.86 ± 0.04 ^a^	0.85 ± 0.05 ^a^	0.99 ns
16	α-bisabolol oxide B	1658	1656	OS	26.59 ± 0.16 ^a^	30.85 ± 0.19 ^b^	1.16 *
17	α-bisabolene oxide A	1685	1684	OS	12.59 ± 0.11 ^a^	16.98 ± 0.08 ^b^	1.35 *
18	α-bisabolol	1686	1685	OS	0.14 ± 0.01 ^a^	0.20 ± 0.01 ^b^	1.43 *
19	chamazulene	1732	1730	SH	10.45 ± 0.21 ^a^	6.98 ± 0.12 ^b^	0.67 *
20	α-bisabolol oxide A	1749	1748	OS	28.33 ± 0.24 ^a^	33.67 ± 0.11 ^b^	1.19 *
21	(*Z*)-en-yn-dicycloether	1832	1830	O	5.55 ± 0.09 ^a^	0.87 ± 0.02 ^b^	0.16 *
22	(*E*)-en-yn-dicycloether	1841	1839	O	6.35 ± 0.08 ^a^	2.81 ± 0.03 ^b^	0.44 *
23	1,4-dimethyl-7-(1-methylethyl)-azulen-2-ol	1936	1934	OS	nd	0.22 ± 0.01 ^a^	- *
	Total				99.91	99.90	1.00 ns
	Sesquiterpene hydrocarbons				13.71	8.77	0.64 *
	Oxygenated sesquiterpenes				74.11	87.24	1.18 *
	Phenylpropanoids				0.19	0.21	1.11 *
	Others				11.90	3.68	0.31 *

^1^ RI_E_—Experimentally determined Retention Indexes; ^2^ RI_LIT_—Retention Indexes—literature data [38]; ^3^ Chemical class: SH—Sesquiterpene hydrocarbons, OS—Oxygenated sesquiterpenes, PP—Phenylpropanoids, O—Others; nd: not detected. Data are reported as the mean ± standard error (*n* = 3). Differences were evaluated by one-way analysis of variance (ANOVA) completed with Fisher’s Least Significant Difference (LSD) test; Means in the same row marked by different letters (a, b) differ significantly (*p* < 0.05); ns: not significant.

## Supplementary Materials

The following are available online at https://www.mdpi.com/2223-7747/9/10/1286/s1, Figure S1: Chromatogram of peppermint essential oil (noninfested plants), Figure S2: Chromatogram of peppermint essential oil (infested plants), Figure S3: Chromatogram of chamomile essential oil (noninfested plants), Figure S4: Chromatogram of chamomile essential oil (infested plants).
