# Supplementary material for: Infestation of Field Dodder (Cuscuta campestris Yunck.) Promotes Changes in Host Dry Weight and Essential Oil Production in Two Aromatic Plants, Peppermint and Chamomile
Source: Plants (Basel). 2020 Sep 29;9(10):1286. doi: 10.3390/plants9101286 (PMC7600651; doi:10.3390/plants9101286)
Supplement: Supplementary file 1 [file plants-09-01286-s001.pdf]

SUPPLEMENTARY MATERIALS

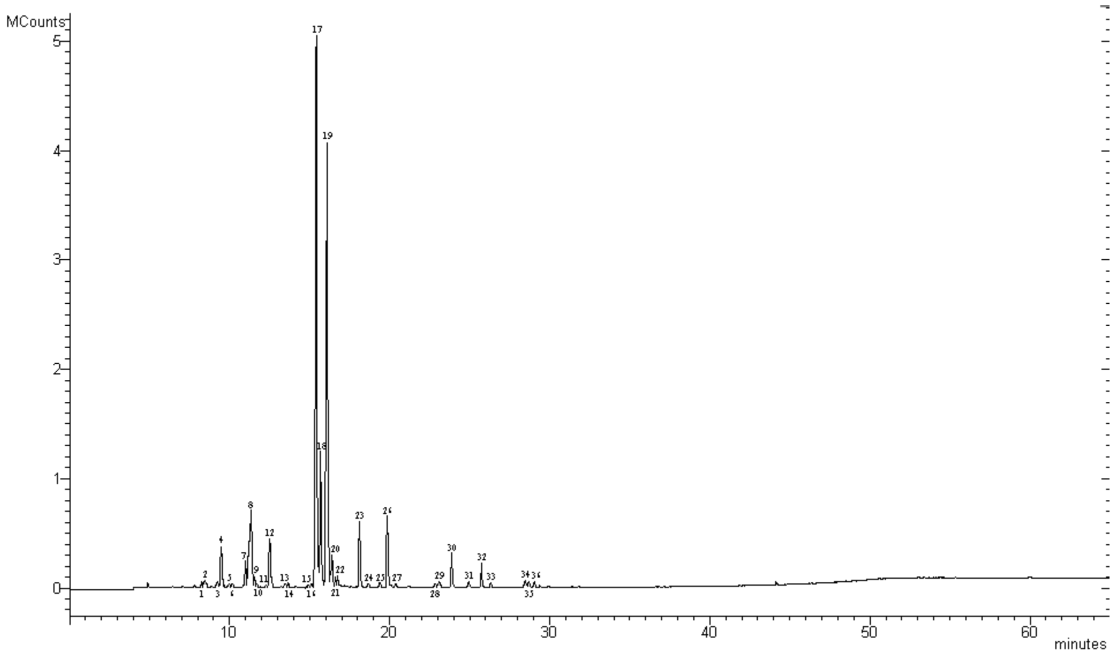

Figure S1. Chromatogram of peppermint essential oil (noninfested plants). 1. α-thujene (Retention time, Rt: 8.201 min); 2. α-pinene (Rt: 8.411 min); 3. Sabinene (Rt: 9.547 min); 4. β-pinene (Rt: 9.665 min); 5. Myrcene (Rt: 10.023 min); 6. 3-octanol (Rt: 10.159 min); 7. p-cymene (Rt: 11.114 min); 8. Limonene+1,8-cineole (Rt: 11.247/11.341 min); 9. (Z)-β-ocimene (Rt: 11.494 min); 10. (E)-β-ocimene (Rt: 11.84 min); 11. γ-terpinene (Rt: 12.216 min); 12. Cis-sabinene hydrate (Rt: 12.502 min); 13. Linalool (Rt: 13.541 min); 14. (2E,4E)-octadienal (Rt: 13.794 min); 15. Trans-sabinol (Rt: 14.935 min); 16. Trans-dihydro-α-terpineol (Rt: 15.133 min); 17. Menthone (Rt: 15.448 min); 18. Menthofuran (Rt: 15.77 min); 19. Menthol (Rt: 16.09 min); 20. 4-terpineol (Rt: 16.218 min); 21. Cis-pinocarveol (Rt: 16.409 min); 22. α-terpineol (Rt: 16.641 min); 23. Pulegone (Rt: 18.253 min); 24. Piperitone (Rt: 18.746 min); 25. Neo-menthyl acetate (Rt: 19.369 min); 26. Menthyl acetate (Rt: 19.98 min); 27. Iso-menthyl acetate (Rt: 20.461 min); 28. (3Z)-hexenyl-(3Z)-hexenoate (Rt: 22.923 min); 29. β-bourbonene (Rt: 23.109 min); 30. β-caryophyllene (Rt: 23.997 min); 31. α-humulene (Rt: 24.987 min); 32. Germacrene D (Rt: 25.831 min); 33. δ-selinene (Rt: 26.286 min); 34. Caryophyllene oxide (Rt: 28.621 min); 35. Globulol (Rt: 28.788 min); 36. Viridiflorol (Rt: 29.029 min). Retention times of n-alkanes used for calculation of RI indices: C9 (Rt: 7.469 min); C10 (Rt: 10.385 min); C11 (Rt: 13.671 min); C12 (Rt: 17.033 min); C13 (Rt: 20.314 min); C14 (Rt: 23.459 min); C15 (Rt: 26.45 min); C16 (Rt: 29.086 min). Used integration parameters were as follows: peak with (sec) - 4.0, slope sensitivity (S/N) – 40, tangent (%) – 10.

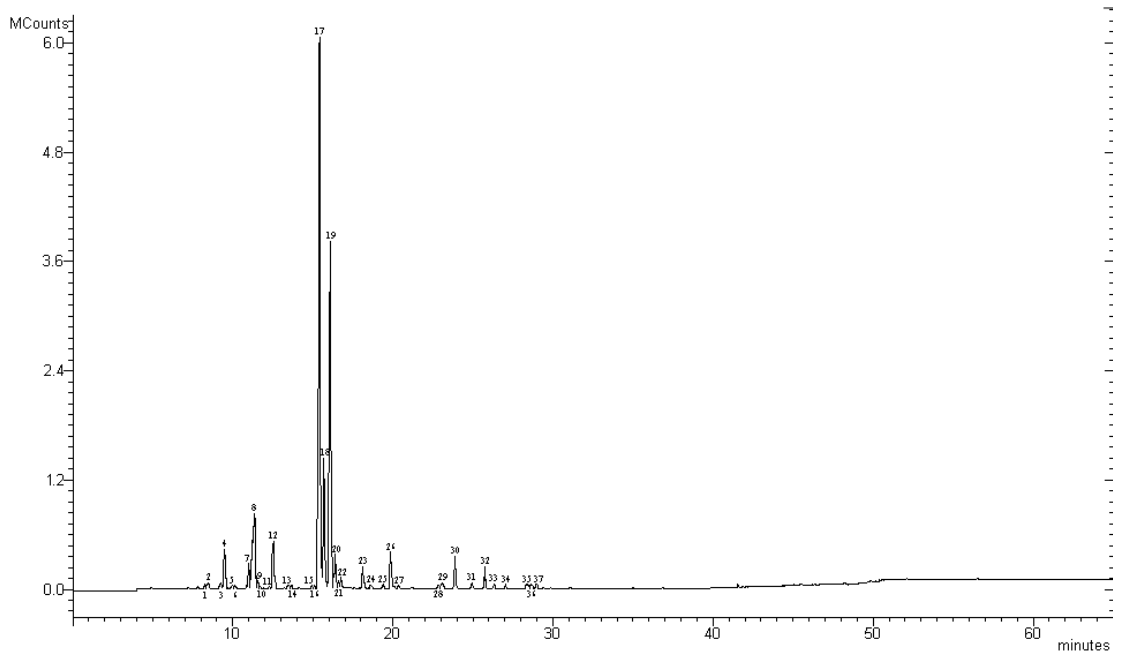

Figure S2. Chromatogram of peppermint essential oil (infested plants). 1. α-thujene (Retention time, Rt: 8.202 min); 2. α-pinene (Rt: 8.413 min); 3. Sabinene (Rt: 9.549 min); 4. β-pinene (Rt: 9.667 min); 5. Myrcene (Rt: 10.025 min); 6. 3-octanol (Rt: 10.163 min); 7. p-cymene (Rt: 11.117 min); 8. Limonene+1,8-cineole (Rt: 11.249/11.344 min); 9. (Z)-β-ocimene (Rt: 11.497 min); 10. (E)-β-ocimene (Rt: 11.844 min); 11. γ-terpinene (Rt: 12.219 min); 12. Cis-sabinene hydrate (Rt: 12.505 min); 13. Linalool (Rt: 13.544 min); 14. (2E,4E)-octadienal (Rt: 13.797 min); 15. Trans-sabinol (Rt: 14.94 min); 16. Trans-dihydro-α-terpineol (Rt: 15.14 min); 17. Menthone (Rt: 15.450 min); 18. Menthofuran (Rt: 15.774 min); 19. Menthol (Rt: 16.092 min); 20. 4-terpineol (Rt: 16.222 min); 21. Cis-pinocarveol (Rt: 16.413 min); 22. α-terpineol (Rt: 16.644 min); 23. Pulegone (Rt: 18.253 min); 24. Piperitone (Rt: 18.746 min); 25. Neo-menthyl acetate (Rt: 19.37 min); 26. Menthyl acetate (Rt: 19.98 min); 27. Iso-menthyl acetate (Rt: 20.462 min); 28. (3Z)-hexenyl-(3Z)-hexenoate (Rt: 22.925 min); 29. β-bourbonene (Rt: 23.11 min); 30. β-caryophyllene (Rt: 23.999 min); 31. α-humulene (Rt: 24.99 min); 32. Germacrene D (Rt: 25.833 min); 33. δ-selinene (Rt: 26.288 min); 34. δ-cadinene (Rt: 27.023 min); 35. Caryophyllene oxide (Rt: 28.622 min); 36. Globulol (Rt: 28.79 min); 37. Viridiflorol (Rt: 29.03 min). Retention times of n-alkanes used for calculation of RI indices: C9 (Rt: 7.469 min); C10 (Rt: 10.385 min); C11 (Rt: 13.671 min); C12 (Rt: 17.033 min); C13 (Rt: 20.314 min); C14 (Rt: 23.459 min); C15 (Rt: 26.45 min); C16 (Rt: 29.086 min). Used integration parameters were as follows: peak with (sec) - 4.0, slope sensitivity (S/N) – 40, tangent (%) – 10.

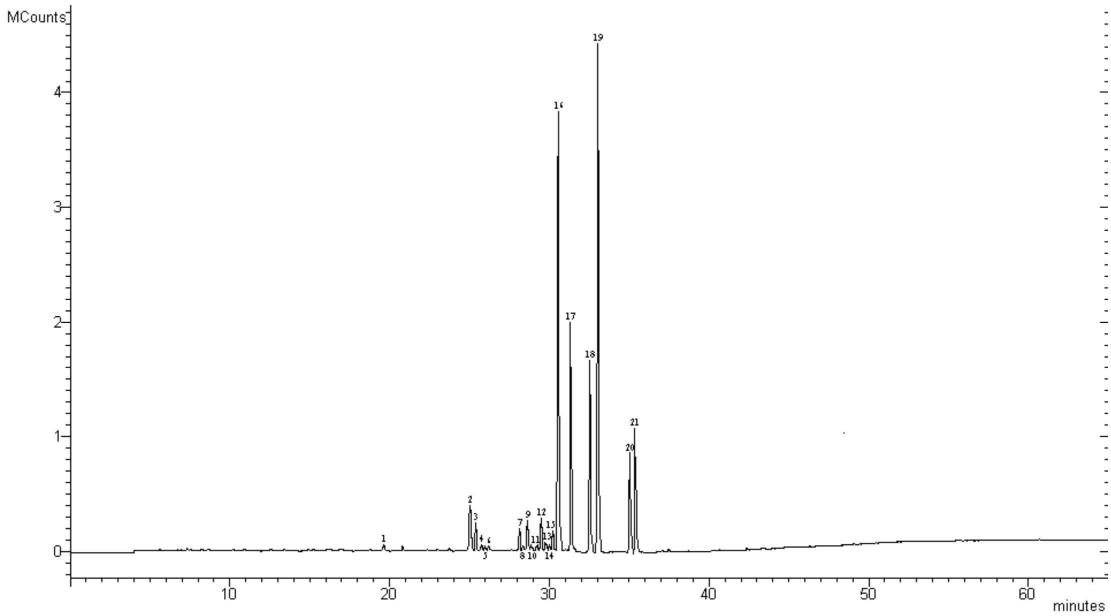

Figure S3. Chromatogram of chamomile essential oil (noninfested plants). 1. (E)-anethole (Retention time, Rt: 19.686 min); 2. (E)-β-farnesene (Retention time, Rt: 25.005 min); 3. Dehydro-sesquicineole (Rt: 25.39 min); 4. γ-muurolene (Rt: 25.809 min); 5. Germacrene D (Rt: 26.022 min); 6. β-selinene (Rt: 26.199 min); 7. (E)-nerolidol (Rt: 28.106 min); 8. (E)-dendrolasin (Rt: 28.303 min); 9. Caryophyllene oxide (Rt: 28.645 min); 10. Salvia-4(14)-en-1-on (Rt: 28.912 min); 11. Guaia-6,10(14)-dien-4β-ol (Rt: 29.319 min); 12. Iso-aromadendrene epoxyde (Rt: 29.477 min); 13. Helifolen-12-al (Rt: 29.597 min); 14. Iso-spathulenol (Rt: 29.953 min); 15. Nerolidol oxide (Rt: 30.129 min); 16. α-bisabolol oxide B (Rt: 30.657 min); 17. α-bisabolene oxide A + α-bisabolol (Rt: 31.375 min); 18. Chamazulene (Rt: 32.611 min); 19. α-bisabolol oxide A (Rt: 33.052 min); 20. (Z)-en-yn-dicycloether (Rt: 35.125 min); 21. (E)-en-yn-dicycloether (Rt: 35.362 min). Retention times of n-alkanes used for calculation of RI indices: C12 (Rt: 17.033 min); C13 (Rt: 20.314 min); C14 (Rt: 23.459 min); C15 (Rt: 26.45 min); C16 (Rt: 29.086 min); C17 (Rt: 31.78 min); C18 (Rt: 34.348 min); C19 (Rt: 36.799 min). Used integration parameters were as follows: peak with (sec) - 4.0, slope sensitivity (S/N) – 40, tangent (%) – 10.

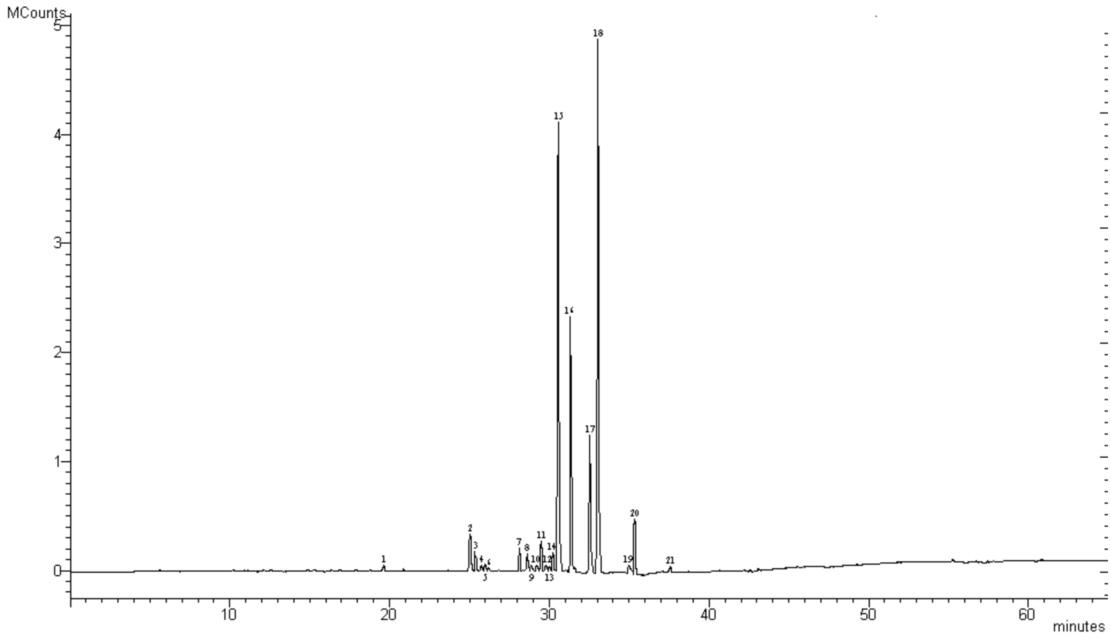

Figure S4. Chromatogram of chamomile essential oil (infested plants). 1. (E)-anethole (Retention time, Rt: 19.687 min); 2. (E)-β-farnesene (Retention time, Rt: 25.007 min); 3. Dehydro-sesquicineole (Rt: 25.39 min); 4. γ-muurolene (Rt: 25.811 min); 5. Germacrene D (Rt: 26.024 min); 6. β-selinene (Rt: 26.201 min); 7. (E)-nerolidol (Rt: 28.106 min); 8. Caryophyllene oxide (Rt: 28.647 min); 9. Salvia-4(14)-en-1-on (Rt: 28.914 min); 10. Guaia-6,10(14)-dien-4β-ol (Rt: 29.320 min); 11. Iso-aromadendrene epoxyde (Rt: 29.478 min); 12. Helifolen-12-al (Rt: 29.596 min); 13. Iso-spathulenol (Rt: 29.956 min); 14. Nerolidol oxide (Rt: 30.131 min); 15. α-bisabolol oxide B (Rt: 30.656 min); 16. α-bisabolene oxide A + α-bisabolol (Rt: 31.376 min); 17. Chamazulene (Rt: 32.612 min); 18. α-bisabolol oxide A (Rt: 33.052 min); 19. (Z)-en-yn-dicycloether (Rt: 35.127 min); 20. (E)-en-yn-dicycloether (Rt: 35.361 min); 21. 1,4-dimethyl-7-(1-methylethyl)-azulen-2-ol (Rt: 37.643 min). Retention times of n-alkanes used for calculation of RI indices: C12 (Rt: 17.033 min); C13 (Rt: 20.314 min); C14 (Rt: 23.459 min); C15 (Rt: 26.45 min); C16 (Rt: 29.086 min); C17 (Rt: 31.78 min); C18 (Rt: 34.348 min); C19 (Rt: 36.799 min); C20 (Rt: 39.142 min). Used integration parameters were as follows: peak with (sec) - 4.0, slope sensitivity (S/N) – 40, tangent (%) – 10.
